#  Giant Macrocystic Lymphatic Malformation in a Neonate

**DOI:** 10.21699/jns.v6i1.383

**Published:** 2017-01-01

**Authors:** Mónica Andrea Pinzón Uresti, Jorge Tadeo Palacios Zertuche, Ana Luisa Carrión García, Julio Cesar Lopez Rodriguez, Juan Pablo Benavides Rodríguez, Ulises de Jesús Garza Luna, Isaías Rodríguez Balderrama

**Affiliations:** Department of Pediatrics “Dr. José Eleuterio González” University Hospital of the School of Medicine of the Universidad Autónoma de Nuevo León, Monterrey, México.

**Dear Sir**

Cystic hygroma or macrocystic lymphatic malformation is a well-known entity and its diagnosis and management is also well described in literature. Rarely its size and location may pose management challenges.[1-3] We managed a case of humongous macrocystic lymphatic malformation of axilla and chest wall in a newborn. At 25 weeks of gestation, a 16cm mass was reported by antenatal ultrasound around left chest wall and arm. At 34.4 weeks of gestation, the baby boy was born by cesarean section. On examination, a 16x16cm mass was noted at the left chest wall with involvement of the skin and subcutaneous tissue; the tumor was soft, mobile and without adherence to deep layers (Fig.1). The baby had respiratory distress because of the location of the tumor and was transferred to the neonatal intensive care unit where ventilatory and hemodynamic support was provided. Plain and contrast magnetic resonance angiography of the neck and arm reported a large mass with characteristics of a hemolymphangioma in the axilla with multiple macrocysts and thin septa having contrast enhancement; the mass did not invade structures or vessels (Fig. 2), surgical resection was performed. An incision was made around the tumor dissecting to the fascia of the pectoral muscle and the serratus anterior. The tumor was gradually dissected to its base to avoid injury to the axillary neurovascular bundle and subsequently removed with skin adnexa. Local skin flaps were made to cover the defect and a closed drainage was placed (Fig. 3). Histopathological study reported a cystic hemolymphangioma. Postoperative recovery was uneventful.

**Figure:**
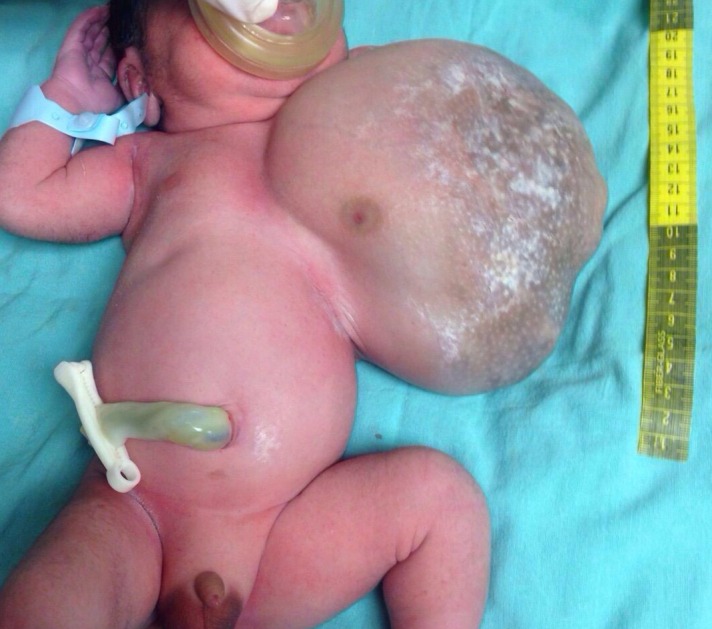
Figure 1: Giant lymphatic malformation.

**Figure:**
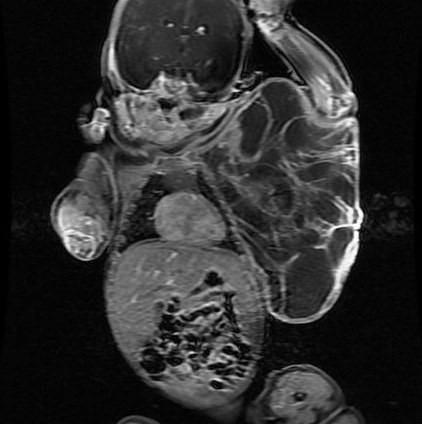
Figure 2: Magnetic resonance angiography that shows mass with characteristics of a hemolymphangioma.

**Figure:**
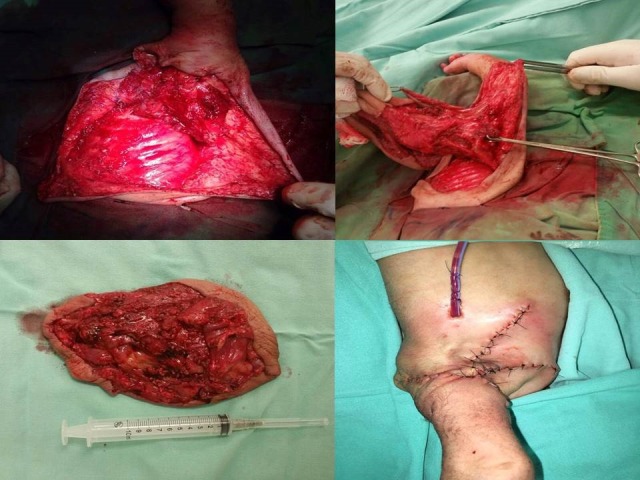
Figure 3: Steps of surgery.

The index case is management challenge. The huge size of the mass led to respiratory distress which was managed by mechanical ventilation in ICU. After initial respiratory and hemodynamic stabilization, the other challenge was excision. Fortunately, the lymphatic malformation was not invading the neurovascular bundle of the axilla and excision went smooth and meticulous.

## Footnotes

**Source of Support:** Nil

**Conflict of Interest:** None
